# Common Protein Biomarkers Assessed by Reverse Phase Protein Arrays Show Considerable Intratumoral Heterogeneity in Breast Cancer Tissues

**DOI:** 10.1371/journal.pone.0040285

**Published:** 2012-07-05

**Authors:** Katharina Malinowsky, Mithu Raychaudhuri, Theresa Buchner, Sabrina Thulke, Claudia Wolff, Heinz Höfler, Karl-Friedrich Becker, Stefanie Avril

**Affiliations:** Department of Pathology, Technische Universität München, Munich, Germany; Health Canada, Canada

## Abstract

Proteins are used as prognostic and predictive biomarkers in breast cancer. However, the variability of protein expression within the same tumor is not well studied. The aim of this study was to assess intratumoral heterogeneity in protein expression levels by reverse-phase-protein-arrays (RPPA) (i) within primary breast cancers and (ii) between axillary lymph node metastases from the same patient. Protein was extracted from 106 paraffin-embedded samples from 15 large (≥3 cm) primary invasive breast cancers, including different zones within the primary tumor (peripheral, intermediate, central) as well as 2–5 axillary lymph node metastases in 8 cases. Expression of 35 proteins including 15 phosphorylated proteins representing the HER2, EGFR, and uPA/PAI-1 signaling pathways was assessed using reverse-phase-protein-arrays. All 35 proteins showed considerable intratumoral heterogeneity within primary breast cancers with a mean coefficient of variation (CV) of 31% (range 22–43%). There were no significant differences between phosphorylated (CV 32%) and non-phosphorylated proteins (CV 31%) and in the extent of intratumoral heterogeneity within a defined tumor zone (CV 28%, range18–38%) or between different tumor zones (CV 24%, range 17–38%). Lymph node metastases from the same patient showed a similar heterogeneity in protein expression (CV 27%, range 18–34%). In comparison, the variation amongst different patients was higher in primary tumors (CV 51%, range 29–98%) and lymph node metastases (CV 65%, range 40–146%). Several proteins showed significant differential expression between different tumor stages, grades, histological subtypes and hormone receptor status. Commonly used protein biomarkers of breast cancer, including proteins from HER2, uPA/PAI-1 and EGFR signaling pathways showed higher than previously reported intratumoral heterogeneity of expression levels both within primary breast cancers and between lymph node metastases from the same patient. Assessment of proteins as diagnostic or prognostic markers may require tumor sampling in several distinct locations to avoid sampling bias.

## Introduction

Various proteins are established as diagnostic and prognostic biomarkers in breast cancer, including estrogen and progesterone receptor status, human epidermal growth factor receptor 2 (HER2) and E-Cadherin [1]. Novel proteins continue to be assessed as potential therapeutic targets and predictive biomarkers. However, intratumoral heterogeneity of protein expression within a primary tumor can pose a challenge when using smaller tumor samples such as core needle biopsies and has not yet been comprehensively studied.

Our goal was to investigate the intratumoral heterogeneity of proteins with clinical relevance to breast cancer, either predictive markers for therapies targeting HER2 [2] or prognostic markers including HER2, estrogen and progesterone receptors [1], E-Cadherin [3], and uPA and PAI-1 [4], [5]. To comprehensively assess protein heterogeneity we further included proteins connected to these candidates via signaling pathways. Thus 15 additional proteins were analyzed belonging either to the same protein family as our candidate proteins (EGFR, HER3, HER4, pPDGFR and VEGFR) or involved in downstream signaling of the candidate molecules (Akt, ERK, FAK, GSK3β, ILK, Integrin αV, PI3K, p38, PTEN and STAT3). In a recent study we demonstrated that several of these proteins are correlated with uPA and PAI-1 expression in primary breast cancers and might be important for uPA and PAI-1 mediated tumor growth and migration [6]. The expression of uPA was correlated with expression of ER and the Stat3/ERK pathway while PAI-1 was associated with Akt signaling and regulation of the HER family. As phosphorylated proteins are often activated proteins we also assessed pAkt, p1086EGFR, p1148EGFR, pER, pERK, pGSK3β, pHer2, pHer3, pPDGFR, pp38, pPR, pPTEN, p727STAT3 and p705STAT3.

The overall goal of this study was to assess the level of heterogeneity of protein expression in breast cancer specimens by analyzing 35 target proteins including 15 phosphorylated proteins representing the HER2, EGFR, and uPA/PAI-1 signaling pathways relevant to breast cancer.

For the analysis of large numbers of samples and target proteins as applied in this study, conventional immunoblot methodology is not suitable as one would need more than 3500 Western blot lanes to conduct a single analysis of all samples and antibodies in our study. The reverse-phase-protein-array (RPPA) is a new approach that allows the simultaneous analysis of multiple samples for the expression of several proteins under the same experimental conditions [7], [8]. RPPA technology also allows analysis of proteins in triplicates and serial dilutions thus enabling reliable quantitative detection of protein expression in the samples. RPPA has widely demonstrated its feasibility for the analysis of cryo-preserved clinical samples [9]–[11]. More recently our group could show that RPPA technology also reliably allows the analysis of formalin-fixed and paraffin-embedded patient samples [12], [13] and is an adequate tool to address protein heterogeneity within such samples.

The aim of this study was i) to determine the intratumoral heterogeneity of 35 proteins representing the HER2, EGFR, and uPA/PAI-1 signaling pathways in large (≥3 cm) primary breast carcinomas, and ii) to identify differences in protein expression levels between axillary lymph node metastases from the same patient. In addition, we assessed the differential protein expression with regard to clinicopathologic parameters and its dependence on the influence of sampling bias with respect to the number of samples taken from each primary tumor.

## Materials and Methods

### Tissue Samples

A total of 106 tissue samples from 15 patients with large (≥3 cm) primary invasive breast cancer with or without associated lymph node metastases were studied. Exclusion criteria were known distant metastases and prior chemo-, hormone-, or radiotherapy. Written informed consent was obtained from all patients and the study protocol was approved by the local institutional review board (Ethikkommission der Fakultät für Medizin der Technischen Universität München).

Ten (67%) of 15 patients had invasive ductal carcinomas, four (26%) invasive lobular, and one (7%) was a mixed ductulo-lobular subtype. For all 15 cases, 2–3 tissue samples were taken each from the peripheral tumor zone defined as the 5 mm peripheral margin, the central zone defined as the 10 mm spherical center, and the intermediate zone between periphery and center. All tissue samples had a size of 5–10 mm side length and 2–4 mm thickness, and the distance between individual samples was >5 mm. In addition, 2–5 axillary lymph node metastases were available in 8 cases. In 7 cases primary tumor tissue without the presence of lymph node metastases was available. Tissue samples were embedded in paraffin according to standard procedures following fixation for 18–24 hours in 10% neutral buffered formaldehyde. H&E stained sections of all paraffin-embedded samples were reviewed to characterize the histological subtype, percentage of viable invasive tumor cells, fibrosis or necrosis, and percentage of inflammatory cells. In addition, information on immunohistochemical expression of ER, PR, and HER2 was obtained for all cases.

All samples showed a tumor cellularity of >70% and <10% inflammatory cells or <10% residual lymphocytes in lymph node metastases. There were no significant differences in epithelial tumor cell content and tumor stroma or inflammatory cell infiltrates between samples from the same patient.

### Protein Extraction

All tissue samples from the same patient (primary tumor and lymph nodes) were processed at the same time. Protein extraction was performed as previously described [6]. Briefly, FFPE tissue sections were deparaffinized, and proteins were extracted using EXB Plus (Qiagen, Hilden, Germany). Tissue areas of approximately 0.25 cm^2^ from three 10 µm thick sections were processed in 100 µl of extraction buffer. The Bradford protein assay (BioRad, Hercules, California US) was used according to the manufacturer’s instructions to determine protein concentrations. A Western blot probing for β-actin was performed from randomly selected lysates (n = 12) to demonstrate successful protein extraction and suitability for RPPA analysis. All protein lysates produced a clear β-actin band on the Western blot.

### Analysis of Protein Expression by Reverse-phase-protein-arrays (RPPA)

The expression of 35 proteins was determined by RPPA. Antibodies and experimental conditions are summarized in Table S1. RPPAs were generated using the Biorad Calligrapher arrayer according to the manufacturer’s instructions (Biorad, Hercules, California, US). For all lysates and dilutions (undiluted, 1∶2, 1∶4, 1∶8, 1∶16, buffer) 3 replicates were applied onto a nitrocellulose coated glass slide (Grace Bio-Labs, Bend, US) producing 18 data points per sample.

Immunodetection was performed similar to Western blot analysis as previously described [14]. For estimation of total protein amounts, arrays were stained in parallel with Sypro Ruby Protein Blot Stain (Molecular Probes, Eugene, US) according to the manufacturer’s instructions. Further details of the RPPA methodology and its validation has been previously described by Wolff et al. [13]. All antibodies used in this study were validated for specificity by Western blot analysis (Figure S1).

### Reproducibility of Protein Extraction and RPPA

10 randomly selected samples of primary breast cancer (not included in the study collective) were extracted in three independent preparations and applied onto two independent arrays as described above. On both arrays levels of HER2, pHER2, uPA and PAI-1 were determined. The Spearman’s rho correlation coefficient and CV were calculated for consecutive extractions and RPPAs, respectively, to assess the technical reproducibility of both methods.

### Statistical Analysis

Intratumoral heterogeneity as well as the range of protein expression amongst different patients (inter-tumor variation) were assessed using the *coefficient of variation* (CV). The CV, defined as the ratio of the standard deviation to the mean multiplied by 100, provides a relative measure for variation independent of the absolute values, and therefore allows comparing the variation of proteins with different absolute expression levels.

Intratumoral heterogeneity was assessed separately for each protein by calculating the CV of all primary tumor samples from the same patient. The variation within tumor zones was assessed by calculating the CV for each tumor zone separately. The variation between tumor zones was assessed by calculating the CV between the mean values for each tumor zone from each patient. The heterogeneity between different lymph node metastases from the same patient was assessed by calculating the CV of all lymph node samples from one patient. As summary statistic, the root-mean-square (RMS) average of the CVs [15] was calculated including all 15 patients to assess the overall intratumoral heterogeneity for a given protein. The RMS-CV was also used to summarize the overall CV of all proteins for a given tumor zone.

The variation between tumors from different patients was assessed for each individual protein by calculating the CV of mean expression values between the different patients. Results are displayed graphically using box-plots showing the median expression value, 25^th^ and 75^th^ quartiles, whiskers (1.5 times the interquartile range) and outliers for each patient.

The Wilcoxon signed rank and Friedman tests were used to compare CVs for different proteins or compare CVs between different tumor zones and the Mann Whitney test was used to compare protein expression between unrelated sample groups at a two-sided 5% level of significance. The inconsistency statistic I^2^ was used to assess the significance of patient-specific differences in CVs across all 35 proteins. I^2^ describes the percentage of variation in CVs between individual patients which is explained by true heterogeneity rather than chance. Cut-offs of 25%, 50% and 75% are commonly used to describe low, moderate, and high variation [16]. The Spearman rank correlation coefficient (rho) was used to assess bivariate relationship of quantitative parameters. All statistical analyses were performed using IBM Statistics (IBM Corporation, Version 19.0) and Origin software (OriginLab Corporation, Version 8).

## Results

### Technical Reproducibility of Protein Expression Analysis

#### Reproducibility of protein extraction

There was a high reproducibility of protein expression from independent extraction procedures (n = 10 samples, n = 30 replicates) with a CV ≤14% for the 4 exemplary proteins HER2, pHER2, uPA and PAI-1. All pairwise correlations showed a Spearman’s rho ≥0.98 (Table S2).

#### Reproducibility of RPPA

There was a high inter-assay reproducibility of protein expression from two independent RPPA analyses (n = 30 samples, n = 60 replicates) for 4 representative proteins (HER2, pHER2, uPA and PAI-1) with a CV ≤12%. All pairwise correlations showed a Spearman’s rho ≥0.94 (Table S2).

**Figure 1:**
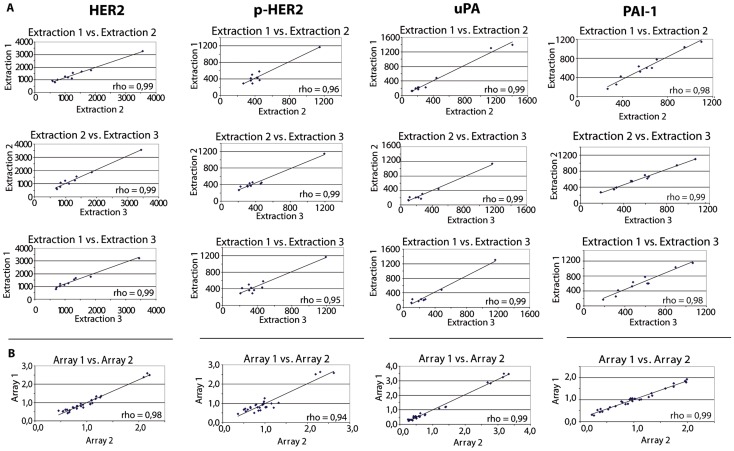
Reproducibility of protein extraction and reverse-phase-protein-arrays. Bivariate correlations of HER2, pHER2, uPA and PAI-1 expression (A) from three independent protein extractions and (B) from two independent RPPA analyses. Graphs are showing the Spearman’s rho for each pairwise correlation.

Correlations are displayed graphically in Figure 1, and pictures of stained replicate arrays shown in Figure S2.

### Intratumoral Heterogeneity of Protein Expression Assessed by RPPA

**Figure 2:**
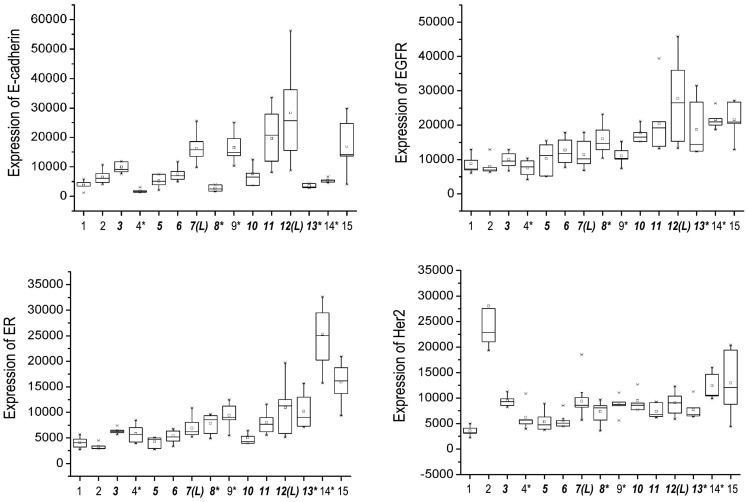
Intratumoral heterogeneity and variation amongst different patients in the expression of 4 exemplary proteins (E-cadherin, EGFR, ER, and HER2) assessed by reverse-phase-protein-arrays. Box plots are showing the median (line within the box), 25^th^ and 75^th^ percentiles, and whiskers are showing 1.5 times the interquartile range; the small box sign is showing the mean, and small cross signs show the maximum and minimum values. ***Italics (bold)***, all cases containing lymph node metastases; * invasive lobular cancers; (L), two largest primary tumors (7.9 and 8.0 cm diameter).

For all 35 proteins considerable intratumoral heterogeneity in expression was observed with a mean CV of 31.0% (range 21.5–43.4%) within samples of primary tumors. A similar extent of heterogeneity was found between axillary lymph node metastases from the same patient, with a CV of 27.2% (range 17.8–34.4%) (Table 1). The extent of intratumoral heterogeneity was different between the 35 individual proteins analyzed (p≤0.001). Figure 2 illustrates the total intratumoral heterogeneity of all cases, including primary tumor and lymph node metastases when available, for the 4 exemplary proteins E-Cadherin, EGFR, ER, and HER2.

**Table 1:** Heterogeneity in the expression of 35 proteins assessed by reverse-phase-protein-arrays.

	Intratumoral heterogeneity [CV]							Variation between patients [CV]				
	Centre	Intermediate	Periphery	Within zones	Between zones	Primary tumor	LN metastases	Centre	Intermediate	Periphery	Primary tumor	LN metastases
Akt	24.3	14.4	20.7	020.3	18.6	21.5	17.8	35.8	43.2	46.7	39.1	46.8
pAkt	26.3	13.6	18.0	018.1	17.0	21.8	18.4	44.6	51.5	50.5	46.6	47.7
E-Cadherin	41.4	23.8	41.3	.36.7	23.4	38.0	30.1	69.3	79.3	90.9	72.8	89.2
EGFR	25.5	27.6	31.1	027.8	25.6	32.0	25.1	37.4	48.1	50.3	39.7	65.4
p1086EGFR	33.7	26.3	29.1	029.0	29.8	34.0	27.7	45.4	61.8	42.6	44.0	43.8
p1148EGFR	29.1	16.7	23.6	023.5	21.1	27.7	23.5	46.0	48.7	58.3	46.5	51.3
ER	29.2	25.8	27.4	026.9	18.8	25.4	25.3	66.1	65.2	69.4	65.3	65.4
pER	22.7	26.0	28.5	025.4	20.8	26.7	24.5	45.6	53.9	66.5	50.4	76.2
ERK	45.4	34.2	32.6	.36.7	21.8	34.3	34.3	76.3	64.1	74.8	68.0	91.1
pERK	25.4	24.0	29.1	022.4	37.7	43.4	29.1	35.7	48.7	105.5	57.1	59.0
FAK	42.8	26.0	33.1	034.6	20.2	34.1	30.8	68.6	48.2	43.5	58.2	55.8
GSK3β	40.5	29.6	29.3	033.4	23.6	37.2	32.2	47.9	55.8	52.9	47.9	69.3
pGSK3β	46.8	32.4	34.6	038.0	26.8	39.7	35.4	93.5	79.3	89.6	80.5	46.4
HER2	34.9	24.7	31.6	030.4	19.2	29.4	26.4	33.8	48.1	49.8	47.5	102.2
pHER2	19.1	31.4	34.8	028.7	20.1	32.1	25.9	69.9	61.8	41.3	74.7	86.3
HER3	33.2	27.8	26.9	028.7	21.8	29.4	26.9	43.7	48.7	57.2	45.2	54.6
pHER3	22.9	27.8	25.5	024.7	24.1	27.7	26.0	41.3	65.2	68.8	42.3	66.3
HER4	31.1	15.9	21.9	023.7	17.9	25.1	20.8	65.9	53.9	66.2	68.1	65.7
ILK	38.5	33.2	33.6	034.4	18.6	34.0	32.2	46.4	64.1	73.7	45.5	50.6
Integrin αV	30.4	25.1	27.0	027.0	22.6	29.7	25.4	58.6	60.5	65.3	56.7	66.5
PAI-1	27.0	12.0	22.1	23.0	18.9	25.3	20.3	60.0	53.6	55.4	55.3	77.8
PI3K	40.5	29.3	26.1	031.5	21.6	32.7	24.5	40.2	60.4	49.6	47.4	45.2
pPDGFR	27.1	17.0	20.1	017.6	18.4	22.8	29.2	44.3	80.2	86.7	39.8	44.1
p38	29.7	22.8	23.3	021.2	22.6	25.6	22.1	34.3	49.6	46.5	39.6	48.9
pp38	21.5	22.0	27.6	23.5	22.7	26.7	21.9	37.3	81.0	88.4	39.4	62.2
PR	23.0	18.9	18.8	19.9	30.7	27.8	20.5	78.5	50.8	56.2	66.3	57.9
pPR	30.3	23.8	21.1	024.6	19.7	25.1	24.2	40.0	47.9	50.9	37.0	47.7
PTEN	27.5	26.3	30.7	027.5	19.0	29.2	25.6	36.8	74.8	81.5	37.7	48.9
pPTEN	26.0	25.4	36.1	029.6	27.7	34.0	34.4	43.5	48.8	48.7	39.7	64.5
STAT3	45.7	33.9	28.4	035.7	23.0	37.3	34.2	41.9	55.6	62.3	48.5	39.9
p227STAT3	27.5	32.6	34.4	30.9	26.1	35.5	33.8	101.4	55.7	64.8	104.3	136.0
p705STAT3	18.9	20.9	23.9	.21.1	28.7	30.2	21.8	45.8	56.4	54.7	45.7	77.6
uPA	34.0	30.8	39.1	034.4	25.5	34.2	31.7	94.6	40.4	43.4	88.4	74.4
uPAR	19.4	22.9	27.2	022.8	20.7	24.3	21.2	47.3	51.3	45.0	50.6	70.9
VEGFR	35.5	23.3	31.3	029.8	31.6	36.3	31.9	44.8	44.6	50.7	38.7	76.3
Phospho	28.9	25.7	28.5	26.0	25.3	31.8	27.3	56.1	61.2	68.3	56.7	69.1
Non-phospho	33.5	25.3	29.0	29.4	22.5	30.6	27.1	56.3	56.1	60.1	55.3	66.8
Overall	31.7	25.5	28.8	28.1	23.5	31.0	27.2	56.2	58.2	63.5	55.8	67.8

There was considerable intratumoral heterogeneity in the expression of all 35 proteins analyzed, and even more pronounced variation amongst different patients.

Intratumoral, differences between individual samples of the same primary tumor or between individual lymph node metastases from the same patient.

CV, coefficient of variation given as percentage; Primary tumor, whole tumor including central, peripheral, and intermediate zone; LN, lymph node; Total, total case including all primary tumor and lymph node samples.

Phospho, all phosphorylated proteins combined; Non-phospho, all non-phosphorylated proteins combined;

Overall, all 35 proteins combined.

There was no difference in the extent of heterogeneity between phosphorylated (mean CV 31.8%) and non-phosphorylated (mean CV 30.6%) proteins. Similarly, the heterogeneity observed within one tumor zone (mean CV 28.1%, range 18.1–38%) or between different zones of the same primary tumor (mean CV 23.5%, range 17.0–37.7%) showed no significant difference (Table 1). There was no overall significant correlation between the diameter of the primary tumor (mean 5.4 cm, range 3.0–8.0 cm) and the extent of intratumoral heterogeneity (Spearman’s rho between −0.3 and 0.2 for 31 proteins; p>0.2). The heterogeneity of EGFR, ER, pHER2 and PAI-1 showed a moderate correlation with tumor size which did not reach statistical significance (rho between −0.5 and 0.4; p>0.1). There was no significant correlation between the percentage of tumor cell content (mean 80%, range 70–98%) and the extent of heterogeneity for the majority of proteins (rho between −0.1 and −0.3 for 26 proteins; p>0.2). The heterogeneity of ER, pPTEN and pp38 was significantly correlated with the percentage of tumor cell content (rho = −0.8, −0.6 and −0.5; p = 0.001, 0.01 and 0.05, respectively). The heterogeneity of EGFR, both pEGFRs, pErk, pHER3 and pSTAT3 showed a moderate correlation with tumor cell content which did not reach statistical significance (rho between −0.4 and −0.5; p>0.06).

There were moderate patient-specific differences in CVs across all 35 proteins with a total variation of 52% (I^2^) between patients.

### Variation of Protein Expression between Different Patients

All 35 proteins showed a very high variation amongst different patients with a mean inter-tumor CV of 51.4% (range 29.3–98.3%) in primary tumor samples and 65.0% (range 39.7–145.5%) in lymph node metastases. There was no significant difference between phosphorylated (mean CV 56.7%) and non-phosphorylated proteins (mean CV 55.3%) (Table 1). The total variation of protein expression amongst different patients is illustrated for 4 exemplary proteins (E-Cadherin, EGFR, ER, and HER2) in Figure 2.

### Differential Protein Expression According to Tumor Subtype, Tumor Stage, Grade, and Hormone Receptor Status

Potential associations of protein expression with clinicopathologic parameters were assessed using the mean expression value assessed by RPPA for each case.

Lobular primary breast cancers (n = 4) showed significantly higher expression of pGSK3β, p727STAT3, and uPA compared to ductal carcinomas (p≤0.03). E-Cadherin showed significantly lower expression in lobular compared to ductal carcinomas.

Estrogen receptor positive tumors (as assessed by immunohistochemistry) showed significantly higher expression of pGSK3β, uPA, PAI-1, and HER4 compared to ER negative tumors (p≤0.03).

Higher stage (T3 and T4) breast cancers showed significantly higher expression of pERK compared to lower stage (T2) tumors (p = 0.02).

Moderately differentiated (G2) tumors showed significantly higher expression of ER, PR, Her4, uPA, PAI-1, and p727STAT3 compared to poorly differentiated (G3) tumors (p≤0.03).

No significant differential protein expression was observed with regard to lymph node status, tumor size (</>5 cm), and immunohistochemical HER2 status.

### Loss of Significance for Differential Protein Expression When Using Single Samples per Case

To illustrate the relevance of multiple sampling and a potential sampling bias, we assessed associations of protein expression with clinicopathologic parameters when taking single samples per case instead of the mean expression values. Single samples with extreme expression values (highest and lowest alternating) by RPPA were retrospectively chosen for each case.

Subsequently, no significant differential protein expression was observed between patient groups according to tumor subtype, immunohistochemical hormone receptor status, immunohistochemical HER2 status, stage, grade, tumor size (</>5 cm) and lymph node status.

## Discussion

Considerable intratumoral heterogeneity was observed for both common and novel protein biomarkers of breast cancer signaling pathways, including HER2, uPA/PAI-1 and EGFR signaling. All 35 proteins studied by reverse phase protein microarrays (RPPA) showed similar heterogeneity with a mean coefficient of variation (CV) of 31.0% (range 21.5–43.4%) within primary breast cancers and 34.7% (range 9.5–79.3%) within different lymph node metastases from the same patient.

Within primary breast cancers we compared several samples from the central, intermediate, and peripheral tumor zones. Interestingly, the extent of heterogeneity was very similar within distinct tumor zones (mean CV 28.1%, range 18.1–38%) and between different zones (mean CV 23.5%, range 17.0–37.7%) suggesting that sampling bias cannot be avoided by taking a single sample from a defined tumor zone but rather sampling one tumor in several distinct locations. Our findings are line with a previous study analyzing the intratumoral heterogeneity of microRNA expression [17].

The extent of intratumoral heterogeneity in protein expression observed in this study is higher compared to previous reports on morphological and molecular heterogeneity of primary breast carcinomas. A possible explanation may be the more extensive systematic sampling of each tumor in several different locations from distinct tumor zones. In contrast, previous studies assessing intratumoral heterogeneity of biomarkers have commonly only analyzed different areas of one tumor section or different core biopsies of the same tumor. A direct comparison of the extent of heterogeneity reported by different studies is hampered by the lack of uniform criteria. Previous studies have provided semiquantitative descriptions of intratumoral heterogeneity, i.e. for expression of hormone receptors and Her2 [18]–[20] or allelic loss and gene amplification [21]–[24] but rarely statistical measures of intratumoral variation.

A possible explanation for the intratumoral heterogeneity in protein expression is variation in the cellular composition of tumor samples. There were no significant differences in epithelial tumor cell content and tumor stroma or inflammatory cell infiltrates among samples from the same patient. Nevertheless, three proteins (ER, pPTEN and pp38) showed a correlation between lower tumor cell content and higher extent of heterogeneity. The presence of different tumor cell clones would be another potential explanation for heterogeneity in protein expression. Previous studies found intratumoral heterogeneity for allelic loss [21], [22] and gene amplification [23], [24]. A thorough analysis of cell clonality including DNA, RNA, and protein analysis would be required to elucidate this hypothesis.

Differences in tumor growth and regional tumor cell proliferation may have contributed to the intratumoral heterogeneity. We previously observed considerable intratumoral heterogeneity in tumor cell proliferation [17] which was similar to the heterogeneity in protein expression detected here (CV 23.0% vs. 31.0%). However, it is unlikely that a single explanation will describe the considerable intratumoral heterogeneity of protein expression observed. Our findings suggest that regional differences in tumor cell proliferation contribute to intratumoral heterogeneity but cannot solely explain the variations found in protein expression in different tumor regions.

There was no overall correlation between the diameter of the primary tumors and the magnitude of intratumoral heterogeneity. Although larger tumors often show more morphological or architectural heterogeneity, such as variation in nuclear grade or tubule formation, we found a comparable heterogeneity of protein expression in tumors ranging from 3–8 cm in diameter. However, we observed a tendency for higher extent of heterogeneity in smaller tumors for few proteins (EGFR, ER, and pHER2).

In comparison, we assessed the variation of protein expression amongst tumors from different patients, which revealed a CV of 51.4% (range 29.3–98.3%) for primary tumor samples and 65.0% (range 39.7–145.5%) for lymph node metastases. Therefore, the intratumoral heterogeneity observed in this study could introduce a significant bias when using only a single sample from tumors. For example, the mean expression of EGFR in the primary tumor of case 4 was significantly lower compared to case 5. Nevertheless, one sample of case 4 showed a higher expression level of EGFR than the lowest of case 5.

To further illustrate the relevance of intratumoral heterogeneity, we assessed associations between protein expression and clinicopathologic parameters when using either all samples per case or just one sample with the lowest or highest expression value. Several proteins including ER, PR, HER4, uPA, PAI-1, and phosphorylated p727STAT3 showed significantly higher expression in moderately differentiated G2 compared to G3 tumors based on mean expression values of all samples for each primary tumor. Interestingly, the significance of this correlation was lost when only one sample was randomly chosen for each primary tumor. Similarly, a significant correlation between higher expression of phosphorylated pGSK3β, uPA, PAI-1, and HER4 in ER positive compared to negative tumors was only observed when using mean expression values of all samples for each primary tumor and lost significance when using only single samples. Significant correlations between protein expression and clinicopathologic parameters were also observed for tumor stage (pERK) and lobular versus ductal subtype (pGSK3β, p727STAT3, uPA, E-Cadherin), all of which lost significance when using only single samples for each primary tumor.

We also assessed the influence of technical variations on quantification of protein expression using a total of 30 technical replicates for protein extraction and 60 replicates for RPPA analyses. We found a high reproducibility of protein measurements from independent extractions (CV≤14%). Similarly, there was a high reproducibility of protein measurements using independent RPPA analyses (CV≤12%). Nevertheless, technical variations may have contributed to some degree to the heterogeneity in protein expression detected in this study.

As mentioned above, an important finding is that the 35 candidate proteins all showed considerable intratumoral heterogeneity although the overall extent of heterogeneity was different between the 35 proteins. It is difficult to estimate if our findings can be extrapolated to other novel proteins relevant to breast cancer. Nevertheless, intratumoral heterogeneity may lead to significant sampling bias when comparing protein expression in tumors from different patients. Intratumoral heterogeneity needs to be taken into account when using protein biomarkers for characterization of different breast cancer subtypes, or prediction of prognosis or response to treatment. In future analyses, the best statistical approach for combining multiple samples from one tumor will depend on the specific study design. Alternatively a practical approach may also be to pool the samples of one case prior to analysis.

Gerlinger et al [25] recently studied 4 metastatic renal cell carcinomas analyzing several regions of the primary tumor and metastatic sites by multiregion sequencing. They observed considerable intratumoral heterogeneity of mutations, with 63 to 69% of all somatic mutations not detectable across every tumor region. In addition, gene-expression signatures of good and poor prognosis were detected in different regions of the same tumor. The authors concluded that a single tumor sample reveals only a minority of genetic aberrations that are present in an entire tumor, and prognostic gene-expression signatures may not correctly predict outcomes if they are assessed from a single tumor region [25].

Limitations of the current study include the number of cases (n = 15) and individual tissue samples (n = 106). An arbitrary cutoff was set at >70% tumor cell content to avoid substantial contamination from non-tumor tissue. We analyzed heterogeneity on a macroscopic level by reverse phase microarrays, and did not assess heterogeneity on a cellular level. The current study assessed only tumors ≥3 cm in diameter. Although our data shows no indication that the extent of intratumoral heterogeneity is generally dependent on the tumor diameter, it is possible that the extent of intratumoral heterogeneity may be different for smaller tumors. An important strength is the systematic and predefined prospective sampling of the tumors in 8–10 different areas, whereas previous studies assessing intratumoral heterogeneity of biomarkers have commonly only analyzed different areas of one tumor section or different core biopsies of the same tumor.

It is important to note that our assessment of protein expression by RPPA provides continuous quantitative measurements which cannot be directly translated to the 2- or 3-tiered immunohistochemical grading system. A direct comparison between RPPA and immunohistochemistry (IHC) has only been performed for few proteins on limited sample numbers [26]–[28]. In 95 breast cancers, Hennessy et al. found a positive correlation between ER and PR levels determined by RPPA and the percentage of positive cells by IHC [28]. Nevertheless, it is important to note that the linear dynamic range of RPPA for detecting differences in protein expression is much larger compared to IHC. Among 64 ER-positive breast cancers as assessed by IHC, RPPA detected a 866-fold difference in ER expression [28]. We previously reported high concordance of HER2 expression measured by RPPA and IHC in breast cancer specimens (94.2%–100%), whereas there was no significant correlation between RPPA and IHC-based determination of hormone receptors [26], [27]. Although we detected considerable intratumoral heterogeneity in quantitative protein expression by RPPA it is unclear how this may be translated to changes between immunohistochemical staining categories. A comprehensive measurement of protein heterogeneity by IHC was beyond the scope of this study and should be addressed in further investigations.

In conclusion, established and novel protein biomarkers of breast cancer including hormone receptors, HER2, uPA/PAI-1, EGFR, pPDGFR, Akt, ERK, PTEN, STAT3 and others, showed considerable intratumoral heterogeneity when assessed by reverse-phase-protein-arrays higher than previously reported for common breast cancer biomarkers [18]–[20]. To avoid sampling bias, assessment of novel breast cancer protein biomarkers for diagnosis or prognosis should be based on primary tumor samples from several different locations, or sampling of several tumor-involved lymph nodes.

## Supporting Information

Figure S1 Validation of antibody specificity by Western blot.
(TIF)

Figure S2 Reproducibility of protein extraction and RPPA.
(TIF)

Table S1 Antibodies and detection conditions used for reverse-phase-protein-arrays.
(DOC)

Table S2 Technical reproducibility of protein extraction.
(DOC)
