# Supplementary figures and images for: Common Protein Biomarkers Assessed by Reverse Phase Protein Arrays Show Considerable Intratumoral Heterogeneity in Breast Cancer Tissues
Source: PLoS One. 2012 Jul 5;7(7):e40285. doi: 10.1371/journal.pone.0040285 (PMC3390380; doi:10.1371/journal.pone.0040285)

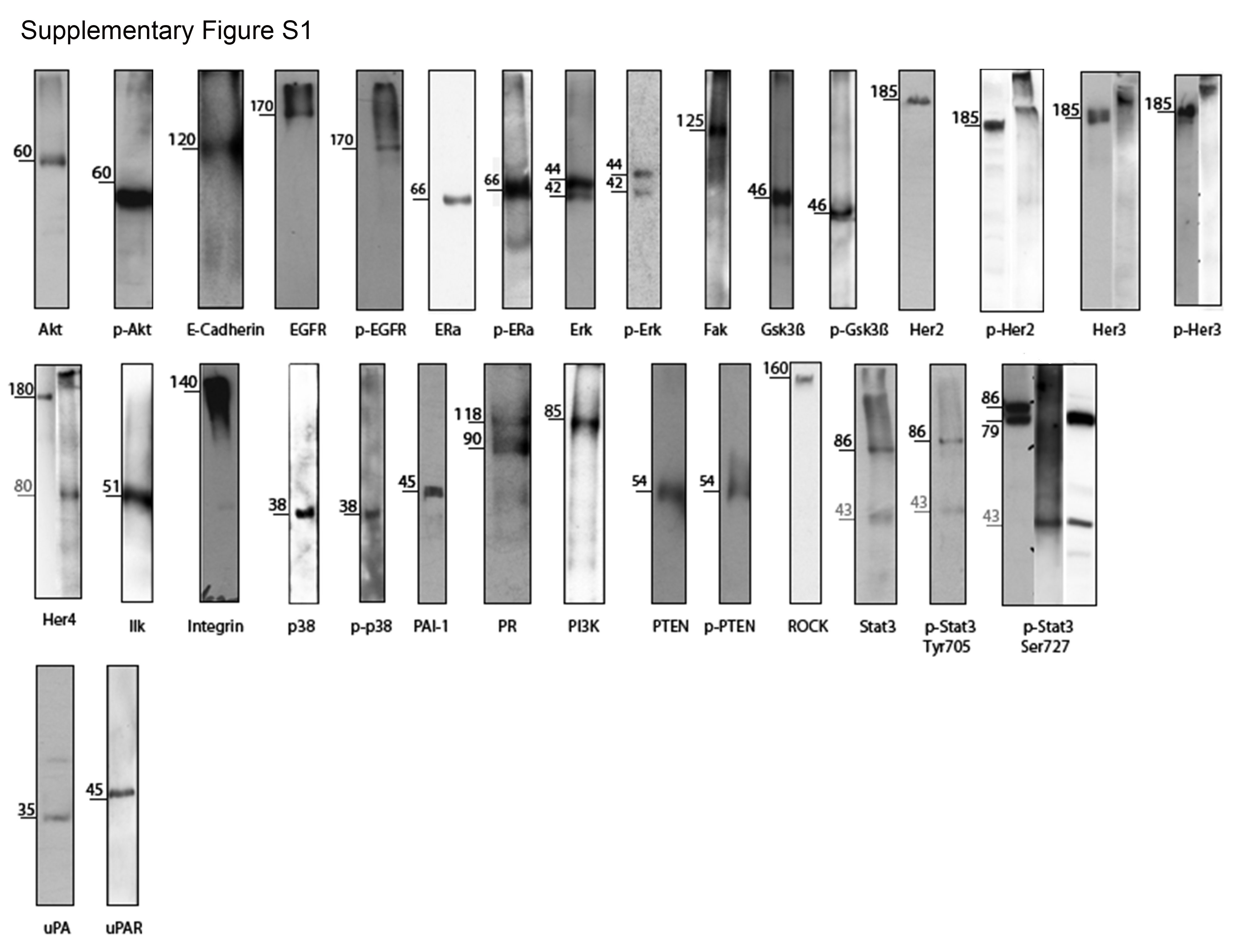

Supplement: Figure S1 — Validation of antibody specificity by Western blot. (TIF) [file pone.0040285.s001.tif]

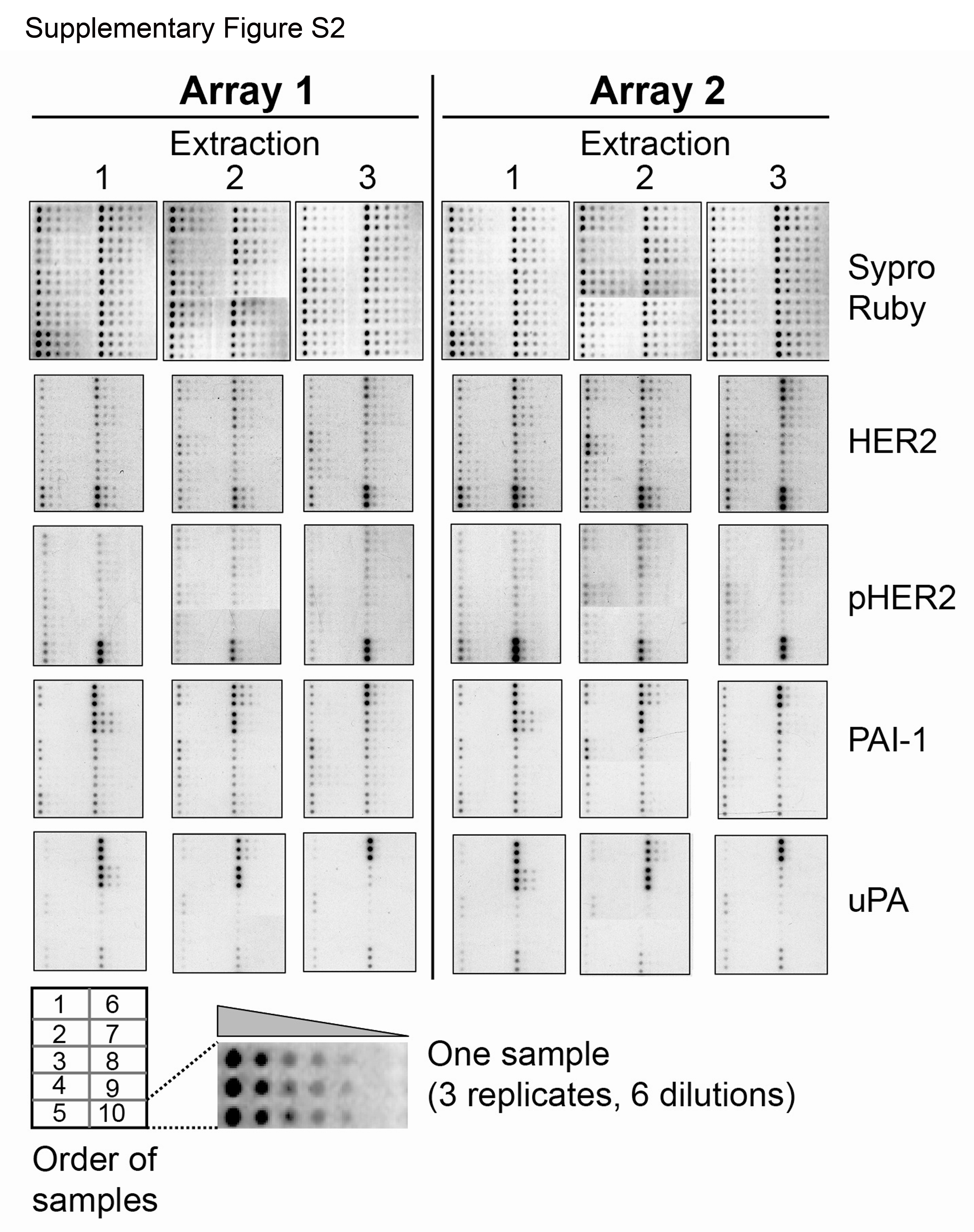

Supplement: Figure S2 — Reproducibility of protein extraction and RPPA. (TIF) [file pone.0040285.s002.tif]
