# Supplementary material for: Common Protein Biomarkers Assessed by Reverse Phase Protein Arrays Show Considerable Intratumoral Heterogeneity in Breast Cancer Tissues
Source: PLoS One. 2012 Jul 5;7(7):e40285. doi: 10.1371/journal.pone.0040285 (PMC3390380; doi:10.1371/journal.pone.0040285)
Supplement: Table S1 — Antibodies and detection conditions used for reverse-phase-protein-arrays. (DOC) [file pone.0040285.s003.doc]

Supplementary Table S1. Antibodies and detection conditions used for reverse phase protein arrays

|  | **Antibody** | **Distributor** | **Dilution** |
| --- | --- | --- | --- |
| AKT | #9272 | Cell signalling, Danvers, US | 1:1000 |
| p^S473^AKT | #9271 | Cell signalling, Danvers, US | 1:1000 |
| E-Cadherin | 610182 | BD Biosciences Pharmingen, San Diego, US | 1:5000 |
| EGFR | #2232 | Cell signalling, Danvers, US | 1:2000 |
| p^Y1086^EGFR | ZMD.504 | Invitrogen, Carlsbad, US | 1:5000 |
| p^Y1148^EGFR | #4404 | Cell signaling, Danvers, US | 1:1000 |
| ERα | #E1629R06SG | DCS, Hamburg, Germany | 1:20 |
| p^S118^ERα | #2511 (16J4) | Cell signalling, Danvers, US | 1:2000 |
| ERK | #9102 | Cell signalling, Danvers, US | 1:1000 |
| p^Y202/204^ERK | #9101 | Cell signalling, Danvers, US | 1:1000 |
| FAK | #3285 | Cell signalling, Danvers, US | 1:2000 |
| GSK3β | #9315 27C10 | Cell signalling, Danvers, US | 1:1000 |
| p^S9^GSK3β | #9336 | Cell signalling, Danvers, US | 1:1000 |
| HER2 | #A0485 | DakoCytomation, Glostrup, Denmark | 1:1000 |
| p^Y1248^HER2 | #44-900 | Invitrogen, Karlsruhe, Germany | 1:1000 |
| HER3 | Ab40627 | Abcam, Cambridge, UK | 1:200 |
| p^Y1289^HER3 | #4791 | Cell signalling, Danvers, US | 1:1000 |
| HER4 | #4795 | Cell signalling, Danvers, US | 1:1000 |
| ILK | #3856 | Cell signalling, Danvers, US | 1:2000 |
| Integrin αV | #4711S | Cell signalling, Danvers, US | 1:2000 |
| PAI-1 | AHP1100 | Serotec, Oxford, UK | 1:5000 |
| pPDGFR | #4549 | Cell signaling, Danvers, US | 1:500 |
| PGR | #PI633R06 | DCS, Hamburg, Germany | 1:20 |
| p^S190^PGR | #3171 | Cell signalling, Danvers, US | 1:1000 |
| PI3K | #4292 | Cell signalling, Danvers, US | 1:1000 |
| PTEN | #9552 | Cell signalling, Danvers, US | 1:2000 |
| p^S380^PTEN | #9551 | Cell signalling, Danvers, US | 1:2000 |
| p38 | #9212 | Cell signalling, Danvers, US | 1:1000 |
| p^T180/Y182^p38 | 12F8, #4631 | Cell signalling, Danvers, US | 1:1000 |
| Stat3 | #4940 | Cell signalling, Danvers, US | 1:1000 |
| p^Y705^Stat3 | #9145 | Cell signalling, Danvers, US | 1:1000 |
| p^S727^Stat3 | #9136 | Cell signalling, Danvers, US | 1:1000 |
| uPA | Ab19893 | Abcam, Cambridge, UK | 1:500 |
| uPAR | Sc9793 | Santa Cruz, Santa Cruz, US | 1:2000 |
| VEGFR | #2479 | Cell signaling, Danvers, US | 1:500 |
