# Supplementary material for: Common Protein Biomarkers Assessed by Reverse Phase Protein Arrays Show Considerable Intratumoral Heterogeneity in Breast Cancer Tissues
Source: PLoS One. 2012 Jul 5;7(7):e40285. doi: 10.1371/journal.pone.0040285 (PMC3390380; doi:10.1371/journal.pone.0040285)
Supplement: Table S2 — Technical reproducibility of protein extraction. (DOC) [file pone.0040285.s004.doc]

**Supplementary Table S2. Technical reproducibility of protein expression analysis**

|  | **Protein extraction** | | **RPPA** | |
| --- | --- | --- | --- | --- |
|  | Spearman’s rho | CV | Spearman’s rho | CV |
| HER2 | 0.98 | 11.9 | 0.98 | 7.6 |
| pHER2 | 0.96 | 14.0 | 0.94 | 12.5 |
| uPA | 0.99 | 13.8 | 0.99 | 8.3 |
| PAI-1 | 0.99 | 10.6 | 0.99 | 8.5 |
